# Development of an X-ray real-time stereo imaging technique using synchrotron radiation

**DOI:** 10.1107/S0909049511017547

**Published:** 2011-05-24

**Authors:** Masato Hoshino, Kentaro Uesugi, James Pearson, Takashi Sonobe, Mikiyasu Shirai, Naoto Yagi

**Affiliations:** a Research and Utilization Division, Japan Synchrotron Radiation Research Institute, 1-1-1 Kouto, Sayo, Hyogo 679-5198, Japan; b Monash Center for Synchrotron Science, Monash University, 770 Blackburn Road, Clayton, Victoria 3800, Australia; c Department of Cardiac Physiology, National Cerebral and Cardiovascular Center Research Institute, 5-7-1 Fujishiro-dai, Suita, Osaka 565-8565, Japan

**Keywords:** X-ray stereo imaging, real-time imaging, angiography

## Abstract

An X-ray stereo imaging system with synchrotron radiation was developed at BL20B2, SPring-8. A portion of a wide X-ray beam was Bragg-reflected by a silicon crystal to produce an X-ray beam which intersects with the direct X-ray beam. Samples were placed at the intersection point of the two beam paths. X-ray stereo images were recorded simultaneously by a detector with a large field of view placed close to the sample. A three-dimensional wire-frame model of a sample was created from the depth information that was obtained from the lateral positions in the stereo image. X-ray stereo angiography of a mouse femoral region was performed as a demonstration of real-time stereo imaging. Three-dimensional arrangements of the femur and blood vessels were obtained.

## Abstract 2

An X-ray stereo imaging system with synchrotron radiation was developed to perform real-time stereo imaging and stereo angiography.

## 1. Introduction

X-ray imaging techniques using synchrotron radiation are generally divided into two categories: one is the three-dimensional imaging technique based on X-ray computed tomography (CT) and the other is two-dimensional radiography which can be performed in real time. In the case of the X-ray micro-CT measurement, transmission images are acquired at multiple angles while rotating a sample and used for the reconstruction of sectional images (Uesugi *et al.*, 2001 ▶). In recent years a fast X-ray micro-CT measurement technique has been developed at SPring-8 using the high-intensity X-ray radiation from an undulator source and a fast-readout detector (Uesugi *et al.*, 2006 ▶). However, it is still difficult to observe a dynamical morphological change of a sample, since the structure of the sample must not change during the 180° rotation. On the other hand, real-time X-ray imaging in the millisecond range has been performed with synchrotron radiation for angiography over many years (Mori *et al.*, 1996 ▶; Dix *et al.*, 2003 ▶; Bertrand *et al.*, 2005 ▶; Schwenke *et al.*, 2007 ▶; Shirai *et al.*, 2009 ▶; Yuan *et al.*, 2010 ▶). More recently, X-ray phase-contrast imaging has been applied to observe the lung and its airways just after birth (Hooper *et al.*, 2009 ▶). To observe the penetration of air into the lung, real-time recording of projection images at millisecond time resolution is required. Although real-time imaging is suitable for studying physiological functions in animals and morphological changes of other materials, it provides only projection images that are often difficult to interpret because of the overlap of objects in the sample.

Here we present a new technique for real-time imaging with three-dimensional information using stereo projections. Recently some studies on X-ray stereo imaging using synchrotron radiation have been reported (Gleber *et al.*, 2009 ▶; Siegbahn *et al.*, 2011 ▶). In those cases, however, stereo images were not obtained simultaneously, but acquired individually by rotating a sample. In the present study, stereo projections are detected simultaneously to achieve real-time stereo imaging. Although complete information on the three-dimensional object cannot be obtained, it can provide three-dimensional information of dynamical phenomena with a time scale of milliseconds. We also present a demonstration of real-time stereo (biplane) angiography.

## 2. Experimental set-up of the stereo imaging system

**Figure 1:**
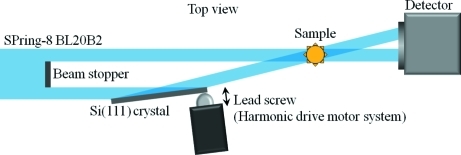
Schematic diagram of the experimental set-up for X-ray stereo imaging.

Two X-ray beams which intersect each other at the sample position (isocenter) must be generated to achieve stereo imaging. A schematic diagram of the optical set-up for stereo imaging is shown in Fig. 1 ▶. This system was constructed at BL20B2, SPring-8. It is a medium-length beamline employing a bending-magnet source, and a monochromatic X-ray beam is extracted by a Si(111) double-crystal monochromator (Goto *et al.*, 2001 ▶). In the experimental hutch located at 200 m from the source a monochromatic X-ray beam with a maximum beam width of 300 mm is available. In the present experiment, 15 keV monochromatic X-rays were used. To realise intersection of two X-ray beams at the sample position, part of the wide incident beam was Bragg-reflected horizontally by a Si(111) crystal at 100 mm from the direct beam. To ensure adequate width of the reflected beam, a 200 mm × 100 mm (thickness 5 mm) rectangular silicon crystal was used. It was set to satisfy the Bragg condition as shown in Fig. 1 ▶. The angle between the two beams was 15.15°.

The X-ray detector consisted of a visible-light conversion unit (Beam Monitor 5; Uesugi *et al.*, 2011 ▶) and a fast-readout electron multiplying charge-coupled device (EM-CCD) camera (C9100-02, 1000 × 1000 pixels, 8µm per pixel, Hamamatsu Photonics KK, Japan). The incident X-ray beam is converted into visible light by a powder P43 (Gd_2_O_2_S:Tb) scintillator of thickness 50 µm. A relay lens of focal length 200 mm transmits visible light to a second relay lens of focal length 35 mm installed in front of the EM-CCD to form a demagnified image on the CCD chip. Since the demagnification of the relay lens system is ×0.175, the effective pixel size and the field of view of the detector was 45 µm and 45 mm × 45 mm, respectively. Since the camera could read out full-frame images at 30 Hz and the decay time of the scintillator was approximately 1 ms, it was possible to capture dynamical phenomena of live animals. In this stereo imaging system the direct and reflected beams were both observed by the same detector. Since the optical axis of the reflected beam was not perpendicular to the scintillator, as shown in Fig. 1 ▶, the width of the reflected beam image was digitally corrected to compensate for the oblique-incidence effect. Since the detector was set normal to the direct X-ray beam, the reflected beam entered the scintillator of the detector obliquely. However, the difference between the effective thickness of the scintillator for the direct and reflected beams was only 3%, which was too small to affect the spatial resolution of the detector in the present experiment.

**Figure 2:**
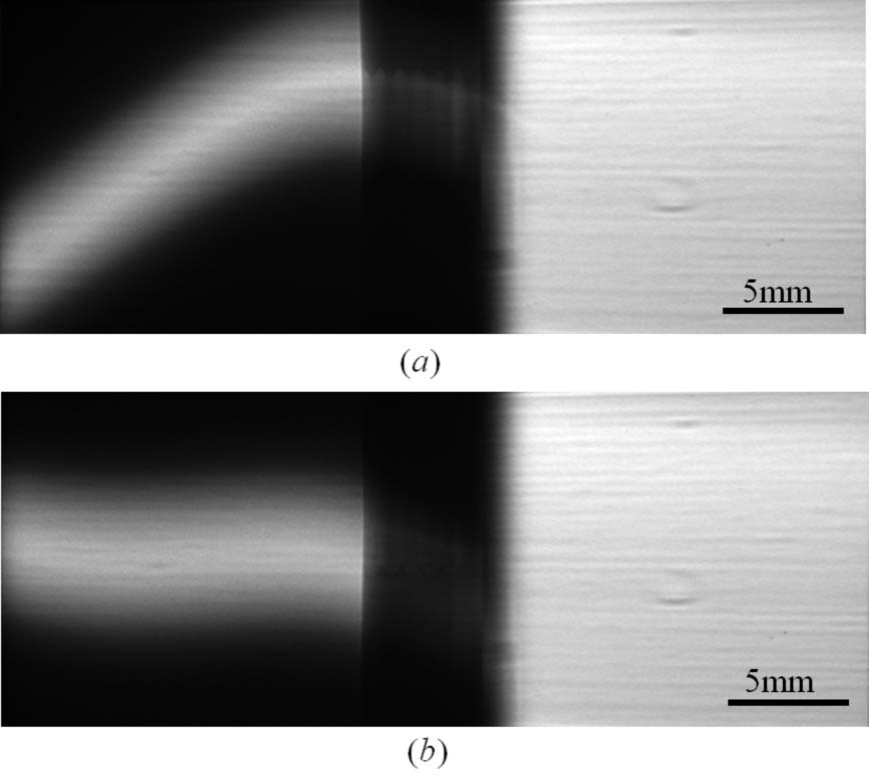
(*a*) Two X-ray beams without a sample. Left: reflected beam. Right: direct beam. (*b*) Two X-ray beams after correction for the oblique shape of the reflected beam.

Stereo X-ray images without a sample are shown in Fig. 2(*a*) ▶. The right-hand image is the direct beam and the left side is the reflected beam. The reflected beam is vertically narrower than the direct beam and has an oblique shape because, within the vertical energy dispersion of the monochromator and both the vertical and horizontal angular spread of the beam, only X-rays with an energy and angle that satisfy the Bragg condition of the silicon crystal are reflected. The unusual shape also arises from deformation of the first silicon crystal in the upstream monochromator which is subject to a high heat load. The height of the reflected beam is approximately 5 mm full width at half-maximum whilst the width is 15 mm, which is determined by the acceptance of the silicon crystal. A simple bender system with a pulse-driven motor system (KIG-20M, KOHZU Precision, Tokyo, Japan), which employs a harmonic drive gearing (Harmonic Drive Systems, Tokyo, Japan), was installed on the crystal holder to correct the profile of the reflected beam. The harmonic drive motor system consists of a motor with harmonic gear reduction and lead screw mechanics, and it is possible to push the crystal using the screw with an accuracy of 0.08 µm per pulse. The silicon crystal was bent in the horizontal plane by pushing one end with the screw while keeping the other end fixed. The distance between the center of the crystal and the screw was approximately 100 mm. The reflected beam profile after correction is shown in Fig. 2(*b*) ▶. The excursion of the screw to bend a crystal was 38.4 µm. Although the beam height remained small, the tilt of the beam was greatly improved. The small distortion in the left half is due to strain on the crystal caused by clamping. Since the reflectivity of the silicon crystal was measured to be 79% at 15 keV, aluminium foils were placed in the direct beam path to equalize the intensity of the two X-ray beams. The thickness of the aluminium foils was adjusted according to the X-ray energy used.

## 3. Stereo imaging

**Figure 3:**
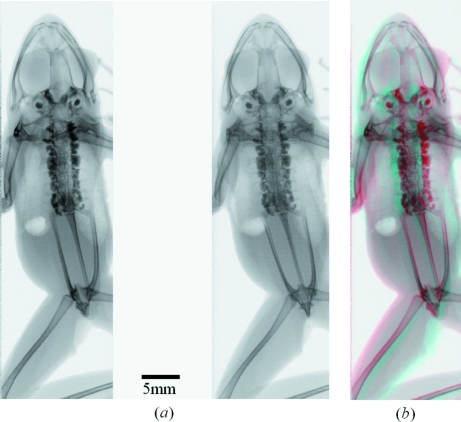
(*a*) X-ray stereo image of the whole body of a frog. This image was digitally combined from sequential images which were acquired by translating the sample vertically. This can be viewed in three dimensions by stereopsis. (*b*) Anaglyph created from the X-ray stereo image.

A living frog (*Rana japonica*) was imaged in stereo. It was not anesthetized but confined in a plastic bag. The bag was attached to a polystyrene plate so that the longitudinal direction of the body became approximately perpendicular to the X-ray beams. The frame rate of the CCD camera was 20 Hz. An X-ray stereo image of the whole body of the frog is shown in Fig. 3(*a*) ▶. Since the vertical field of view of the stereo image was restricted by the beam height of the reflected beam, sequential images were acquired whilst continuously translating the sample vertically. The images were then digitally combined. The frog can be seen in three dimensions by stereopsis. An anaglyph created from the stereo image is also shown in Fig. 3(*b*) ▶.

**Figure 4:**
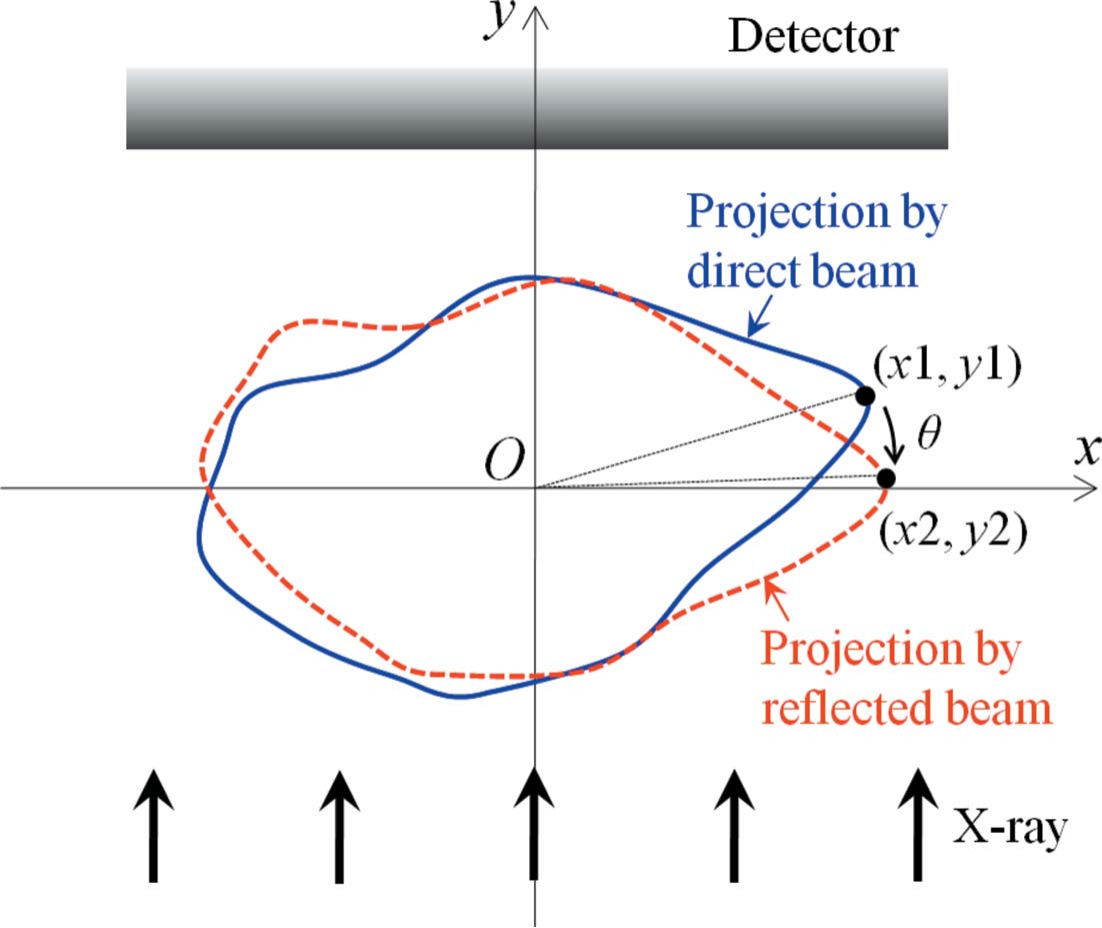
Schematic drawing of the coordinate system for obtaining the depth information of a sample from an X-ray stereo image.

Although complete three-dimensional information of a complex sample such as the biological specimen shown in Fig. 3(*a*) ▶ cannot be obtained from stereo images, the depth information of a simple object can be derived. As shown in Fig. 4 ▶, we assume that stereo images are two projection images of the sample at angles 0 and θ. The angle θ is the intersectional angle of the two X-ray beams. The left-side image in the stereograph (Fig. 2 ▶) corresponds to the projection image at a rotational angle of θ. In the coordinate system shown in Fig. 4 ▶ the origin is at the center of the sample and the X-rays are along the *y*-axis. The *z*-axis is the rotation axis that is perpendicular to the *x*–*y* plane. The relation between a given point in the object (*x*1, *y*1) and its rotated point (*x*2, *y*2) is easily represented as
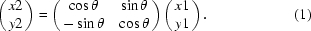
If the *x*-positions at both angles are known from the stereograph, the *y*-position which corresponds to the depth information is derived by 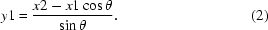
From (2) the three-dimensional coordinate (*x*1, *y*1, *z*1) can be obtained where *z*1 is a common value in the two images.

**Figure 5:**
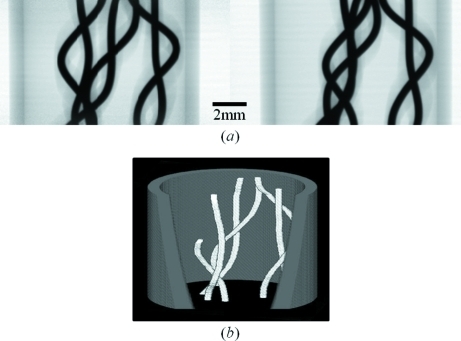
(*a*) X-ray stereo image of six twisted metal wires set in a plastic case. (*b*) Three-dimensional view of the metal wires created from the stereo image in (*a*). The three-dimensional configuration of the plastic case was reconstructed under the assumption that it was a cylinder. It was additionally synthesized into the wire image.

As a demonstration of the three-dimensional analysis, six twisted metal wires in a cylindrical plastic case were imaged. The diameter of the plastic case was 11 mm. An X-ray stereo image is shown in Fig. 5(*a*) ▶. Since the metal wire was covered with a plastic sheath, the diameter, which was approximately 400 µm, was estimated from the stereo image. A three-dimensional view of the metal wires created from the stereo image is shown in Fig. 5(*b*) ▶. In this case the depth position (*y*1) was calculated from two lateral positions (*x*1 and *x*2) which were obtained by tracing the center of each wire manually. The plastic case in Fig. 5(*b*) ▶ was created under the assumption that it had a cylindrical configuration and synthesized into the image of the wires. The three-dimensional structure of the sample was easily recognized (Fig. 5*b* ▶). As mentioned above, however, application of this method to calculate the depth position from the stereo image is limited to samples which can be represented by a wire frame model, and it is required that the corresponding points of the object in the two images can be easily recognized.

## 4. Real-time stereo imaging

**Figure 6:**
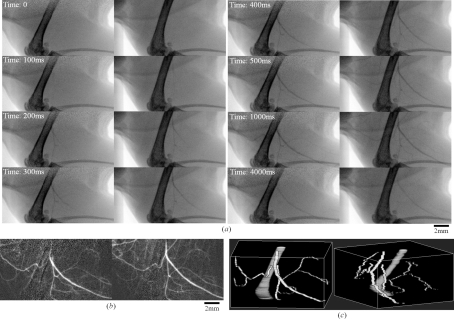
(*a*) X-ray stereo images in angiography. The inset values indicate the elapsed time from the start of the measurement. (*b*) Stereo image of blood vessels obtained by subtracting the stereo image before injection from that after injection (elapsed time:1500 ms). This can be viewed in three dimensions by stereopsis. (*c*) Three-dimensional arrangements of the femur and main blood vessels constructed from X-ray stereo angiography.

To test real-time stereo imaging we performed X-ray stereo angiography of the femoral artery of a mouse (C57BL/6). Although X-ray angiography has usually been performed at an X-ray energy of 33.2 keV, which is just above the *K*-absorption edge of iodine in a contrast agent, to obtain high image contrast, X-ray stereo angiography was performed at an X-ray energy of 15 keV to obtain a large field of view for the reflected beam image. Since the width of the reflected beam depends on the acceptance of the crystal, lower X-ray energy is preferable. At 15 keV, for small animals such as a mouse, adequate X-ray transmission can be obtained across the body, while absorption by iodine is in fact higher than at 33.2 keV. Iomeron-350 (Eisai) was used as a contrast agent, which was administered *via* a 300–400 µm-diameter catheter inserted into the common carotid artery. The injection dose and the rate were 0.1–0.2 ml per injection and 0.1 ml s^−1^, respectively. The frame rate of the CCD camera was 10 Hz. Stereo angiographic images of the femoral artery are shown in Fig. 6(*a*) ▶. The blood vessels can be clearly observed by subtracting the stereo image before injection from that after injection, as shown in Fig. 6(*b*) ▶ (digital subtraction angiography). To visualize the femur and major blood vessels in three dimensions, their depth information was derived from the stereo images in the method described above. Three-dimensional views of the femur and the blood vessels are shown in Fig. 6(*c*) ▶. In these three-dimensional views the cross-sectional shape of the femur and blood vessels were assumed to be a circle. Although the synthesized three-dimensional view shown in Fig. 6(*c*) ▶ is far from the quality of that obtained from X-ray CT, it demonstrated that the time-resolved three-dimensional structure of a complicated sample can be obtained.

## 5. Discussion

In the current stereo imaging system the effective field of view is mainly limited by the reflected beam size from a silicon crystal. The unusual shape of the reflected beam will be improved by modification of the cooling system for the first crystal in the upstream monochromator. A direct water-cooling system is a promising way to improve the cooling capacity instead of the current indirect system. Although the oblique shape of the reflected beam was corrected by deforming a silicon crystal with a simple bender, a prominent deformation of a sample image was not observed. As shown in some stereo images, they can be seen in three dimensions by stereopsis without any distortion corrections except a correction of the image width as mentioned above. The reflected beam image was neither magnified nor demagnified at the CCD plane compared with the direct beam image. It seems that the spatial resolution of the reflected beam image is equal to that of the direct beam image which is just defined by the pixel size of the detector.

Use of stereoscopy in X-ray imaging was proposed more than 100 years ago (Thomson, 1896 ▶) and is still being discussed (Evans & Robinson, 2000 ▶). For medical imaging, biplane X-ray angiography has been used to visualize human coronary vessels (Hoffmann *et al.*, 1995 ▶) and three-dimensional reconstruction techniques from biplane images have been developed (Metz & Fencil, 1989 ▶). However, as far as the authors are aware, this is the first report on dual-beam stereoscopic angiography imaging using synchrotron radiation, while some stereo imaging experiments using synchrotron radiation have been reported (Gleber *et al.*, 2009 ▶; Siegbahn *et al.*, 2011 ▶). In synchrotron radiation imaging experiments, as the samples are mostly experimental animals or non-biological objects, the disadvantage of doubling the dose is less serious than in clinical imaging and the increase in the acquired information is more significant. Although qualitative measurement of coronary arteries is often the purpose of human biplane angiography (Tu *et al.*, 2010 ▶), in small-animal angiography the purpose of stereo imaging is to solve the problem of superposition of blood vessels not specifically coronary vessels.

To increase the amount of information, it is obvious that the number of images should be increased. In the experimental set-up for the stereo imaging tested in this paper, two X-ray beams that intersect at the sample position were generated by reflecting a part of the incident wide X-ray beam. In the future we plan to install silicon crystals at both ends of the wide X-ray beam, thereby creating three X-ray beams, one direct and two reflected beams. When information in the projections from the three directions is combined, identification of the depth information will be easier. The accuracy of the positional information in the depth direction will also be improved. In the present experiment, since there is a double-crystal monochromator in the upstream hutch, the X-ray was monochromatic. With a white beam, it is possible to make multiple beams of different energies with Laue crystals aligned in tandem along the beam. This will be another option to obtain three-dimensional information from multiple projections.

Recently, propagation-based phase-contrast imaging has been used to obtain dynamic information of the lung motion in new-born animals (Hooper *et al.*, 2009 ▶). Another innovation is three-dimensional particle velocimetry using multiple projection angles (Dubsky *et al.*, 2010 ▶). This technique can make use of phase contrast by increasing the distance from the sample to the detector (Irvine *et al.*, 2008 ▶). In the current experiment, stereo images were detected simultaneously by a single detector. This requires that the detector be placed close to the sample which prevents the use of X-ray phase contrast. In order to implement propagation-based phase contrast, two or three detector systems with the same specification need to be used in synchrony to detect stereo or triscopic X-ray images. Since the edge enhancement by propagation-based phase contrast, namely X-ray refractive contrast, depends on the inner shape of the object, there may be some differences in the edge enhancement effect between the images. This may make pattern matching by the numerical calculation more complicated. However, Siegbahn *et al.* (2011 ▶) have already shown an anaglyph stereoscopic X-ray image with propagation-based phase contrast for simple objects. The effectiveness of tomography from propagation phase-contrast images has also been demonstrated (Sera *et al.*, 2005 ▶). If an exact pattern matching is required, a phase-retrieval method from a single projection image will be powerful for recovering an absorption-based image from an edge-enhanced image (Paganin *et al.*, 2002 ▶; Weitkamp *et al.*, 2011 ▶). Such a software development will extend the capabilities of the real-time propagation-based phase-contrast imaging.
